# Efficient Production of Chondrocyte Particles from Human iPSC-Derived Chondroprogenitors Using a Plate-Based Cell Self-Aggregation Technique

**DOI:** 10.3390/ijms252212063

**Published:** 2024-11-10

**Authors:** Shojiro Hanaki, Daisuke Yamada, Tomoka Takao, Ryosuke Iwai, Takeshi Takarada

**Affiliations:** 1 Department of Regenerative Science, Dentistry and Pharmaceutical Sciences, Okayama University Graduate School of Medicine, Okayama 700-8558, Japan; shojiro-hanaki@s.okayama-u.ac.jp (S.H.); dyamada@okayama-u.ac.jp (D.Y.); ttakao@okayama-u.ac.jp (T.T.); 2 Institute of Frontier Science and Technology, Okayama University of Science, Okayama 700-0005, Japan; iwai@ous.ac.jp

**Keywords:** tissue engineering, chondrocyte particles, cartilaginous particles, ExpLBM, hiPSC, chondrocyte

## Abstract

The limited capacity of articular cartilage for self-repair is a critical challenge in orthopedic medicine. Here, we aimed to develop a simplified method of generating chondrocyte particles from human-induced pluripotent stem cell-derived expandable limb-bud mesenchymal cells (ExpLBM) using a cell self-aggregation technique (CAT). ExpLBM cells were induced to form chondrocyte particles through a stepwise differentiation protocol performed on a CAT plate (prevelex-CAT^®^), which enables efficient and consistent production of an arbitrary number of uniformly sized particles. Histological and immunohistochemical analyses confirmed that the generated chondrocyte particles expressed key cartilage markers, such as type II collagen and aggrecan, but not hypertrophic markers, such as type X collagen. Additionally, when these particles were transplanted into osteochondral defects in rats with X-linked severe combined immunodeficiency, they demonstrated successful engraftment and extracellular matrix production, as evidenced by Safranin O and Toluidine Blue staining. These data suggest that the plate-based CAT system offers a robust and scalable approach to produce a large number of chondrocyte particles in a simplified and efficient manner, with potential application to cartilage regeneration. Future studies will focus on refining the system and exploring its clinical applications to the treatment of cartilage defects.

## 1. Introduction

Articular cartilage comprises chondrocytes embedded in an extracellular matrix (ECM), which itself comprises primarily type II collagen (COL2) and proteoglycans. The function of cartilage is to provide shock absorption and facilitate smooth movement of the joint; however, due to its avascular nature, articular cartilage does not regenerate very well after traumatic injury or damage caused by osteoarthritis (OA). OA is a leading cause of disability worldwide, affecting over 527 million people and imposing a growing burden on healthcare systems as populations age [1]. Focal cartilage defects are recognized as potential risk factors for OA, and therapies, such as autologous or allogeneic chondrocyte transplantation are commonly employed to prevent or delay OA progression. Current treatments for small cartilage defects include osteochondral autograft transfer [2], osteochondral allograft transplantation [3], and autologous chondrocyte implantation [4,5]; however, these methods are limited by the need for large quantities of chondrocytes, as well as other constraints that restrict their widespread application. Although these techniques provide temporary symptom relief, they often result in fibrocartilage formation rather than the desired hyaline cartilage—essential for long-term durability and joint function [1].

Recent advancements in cartilage repair, particularly with stem cell-based therapies, seek to overcome these limitations. Human-induced pluripotent stem cells (hiPSCs) offer a promising alternative to traditional sources by providing a potentially unlimited cell supply for chondrogenic differentiation, while also reducing related donor site morbidity. These cells enable more efficient and consistent cartilage regeneration, offering significant potential for clinical applications in joint repair [1].

To address these challenges, we demonstrated previously that hiPSC-derived expandable limb-bud mesenchymal cells (ExpLBM) can be expanded stably under xeno-free conditions while retaining high chondrogenic potential [6]. Importantly, human articular cartilage is ontogenically derived from the neural crest, paraxial mesoderm, and lateral plate mesoderm [7,8,9]. This suggests that lateral plate mesoderm-derived ExpLBM cells could serve as an ideal source for regenerating limb articular cartilage. We also reported previously that ExpLBM cells with high potential for chondrogenic differentiation can be cultured in chondrogenic induction medium in a stirred bioreactor; this process generates large quantities of cartilage tissue [10]. While this method is efficient and facilitates production of large numbers of chondrocyte particles, we found that the number of particles was inconsistent.

Therefore, in the present study, we aim to develop a simplified method for producing an arbitrary number of chondrocyte particles using ExpLBM cells in combination with a plate-based cell self-aggregation technique (CAT).

## 2. Results

### 2.1. Characterization of ExpLBM Cells and Chondrogenic Differentiation

**Figure 1:**
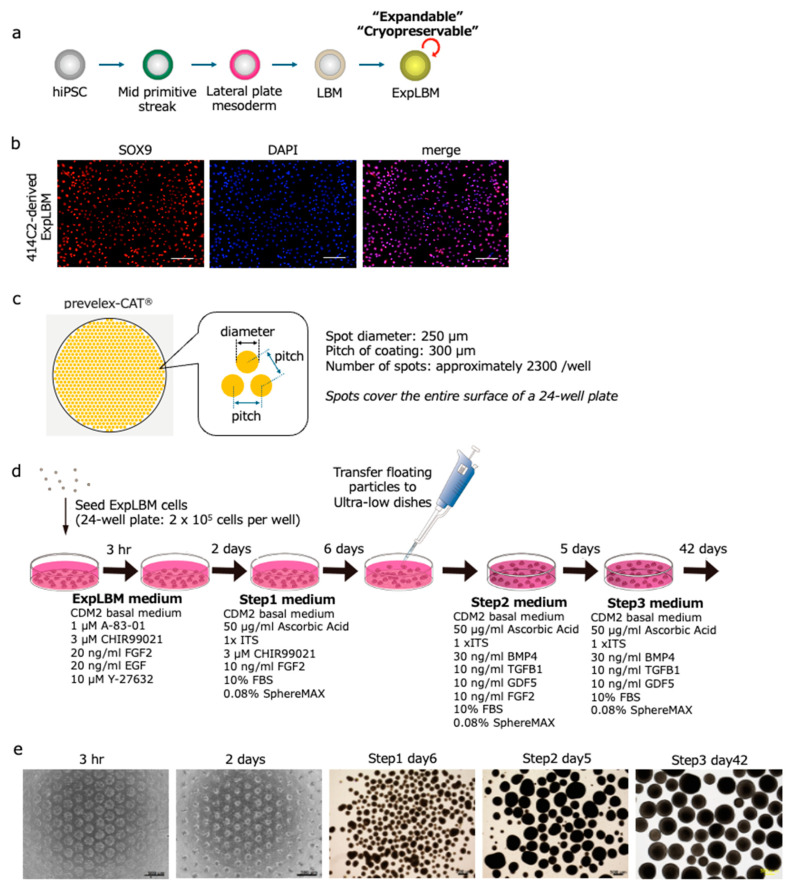
Differentiation of hiPSCs into ExpLBM cells, and fabrication of ExpLBM-derived chondrocyte particles. (**a**) Schematic illustration showing the stepwise differentiation of hiPSCs into ExpLBM cells. (**b**) Immunostaining of 414C2 ExpLBM cells for SOX9 (scale bar: 200 µm). (**c**) CAT plate (prevelex-CAT^®^) showing spot diameter (250 µm), pitch (300 µm), and approximately 2300 spots per well. (**d**) Schematic of the culture protocol for generation of chondrocyte particles from ExpLBM cells using the CAT plate. (**e**) Images showing temporal changes in chondrocyte particle formation, as well as enlargement over time (scale bars: 500 µm).

Previously, we successfully induced development of limb-bud mesenchymal (LBM) cells from hiPSCs, and established a robust method for their expansion [6]. The morphology of 414C2 hiPSCs at each stage of differentiation was clear, showing distinct changes during the differentiation process (Figure 1a). The ExpLBM cells derived from 414C2 hiPSCs expressed SRY-box transcription factor 9 (SOX9) consistently, maintaining nearly 100% positivity across serial passages (Figure 1b), while expression of PRRX1 was also high. Furthermore, flow cytometry analysis demonstrated that these ExpLBM cells exhibited high levels of CD90 and CD140b, both of which are markers indicative of strong chondrogenic differentiation potential.

### 2.2. Fabrication of Chondrocyte Particles Using the CAT Plate System

**Figure 2:**
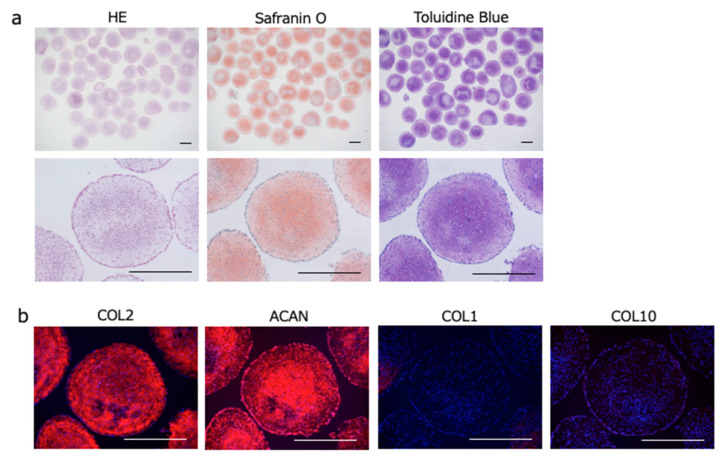
Histological examination of ExpLBM-derived chondrocyte particles. (**a**) Chondrocyte particle sections stained with hematoxylin and eosin (HE), Safranin O, and Toluidine Blue (scale bars: 500 µm). (**b**) Immunohistochemical staining of chondrocyte particle sections for COL1, COL2, COL10, and ACAN (scale bars: 500 µm).

We utilized the CAT developed by our collaborator, Iwai (Institute of Frontier Science and Technology, Okayama University of Science), along with the CAT plate (prevelex-CAT^®^; Nissan Chemical, Tokyo, Japan) to fabricate ExpLBM cell-derived chondrocyte particles (Figure 1c) [11]. Aggregates formed on the CAT plate were subjected to chondrogenic induction during Step 1, Step 2, and Step 3 of the protocol (Figure 1d) [6]. The size of the chondrocyte particles increased gradually and, by Day 42 of Step 3, the desired number of chondrocyte particles of a relatively uniform size was achieved (Figure 1e). Histological analysis showed that the 414C2 ExpLBM-derived chondrocyte particles were embedded in an ECM that was intensely and homogeneously stained with Safranin O and Toluidine Blue (Figure 2a). Immunohistochemistry revealed expression of COL2 and aggrecan (ACAN) by the ECM, with no expression of type I collagen (COL1) or type X collagen (COL10) (Figure 2b). These findings indicate that the ExpLBM-derived chondrocyte particles exhibit characteristics typical of hyaline cartilage.

### 2.3. In Vivo Transplantation and Evaluation of Chondrocyte Particle Efficacy

**Figure 3:**
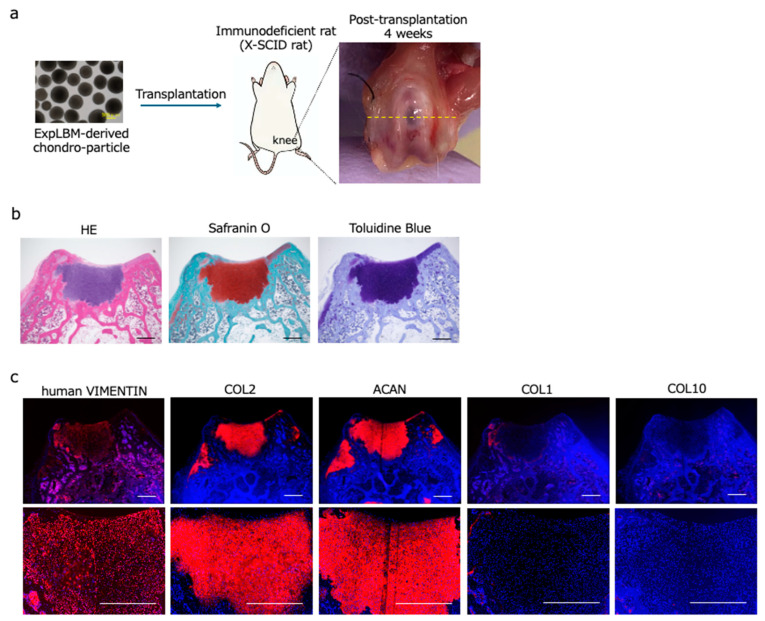
Engraftment of ExpLBM-derived chondrocyte particles in X-SCID rats. (**a**) Schematic showing the procedure used to transplant ExpLBM-derived chondrocyte particles into holes drilled in the knee joints of X-SCID rats. (**b**) Histological analysis of the knee joints 4 weeks post-transplantation. Tissue sections were stained with hematoxylin and eosin (HE), Safranin O, and Toluidine Blue (scale bars: 500 µm). (**c**) Immunohistochemical staining of tissue sections using antibodies specific for human VIMENTIN, COL2, COL1, COL10, and ACAN (scale bars: 500 µm).

To demonstrate their regenerative efficacy in vivo, ExpLBM-derived chondrocyte particles were transplanted into osteochondral defects (1-mm drill hole defects with a depth of 1 mm) created in the knee joint cartilage of rats with X-linked severe combined immunodeficiency (X-SCID) (Figure 3a). Four weeks after transplantation, the 414C2 ExpLBM-derived chondrocyte particles had engrafted successfully into the osteochondral defects, as confirmed by expression of human vimentin. The ExpLBM-derived chondrocytes filling the defects produced an ECM that was stained by Safranin O and Toluidine Blue (Figure 3b). Immunohistochemical analysis revealed the presence of COL2 and ACAN in numerous ECM regions of the regenerated neocartilage, while COL1 and COL10 were restricted to the surface of the neocartilage (Figure 3c).

## 3. Discussion

In this study, we successfully developed a simplified method for generating chondrocyte particles from ExpLBM cells using a plate-based CAT. This method enables production of an arbitrary number of uniformly sized chondrocyte particles. The main advantage of the plate-based CAT system is its ability to facilitate efficient cell aggregation in a controlled manner, thereby producing consistent and scalable quantities of chondrocyte particles. This method is highly efficient compared with conventional culture plate methods due to its capability for large-scale cartilage tissue production [6,12,13,14]. Unlike previous approaches involving a stirred bioreactor, which often encounters limitations in achieving uniformity and consistency in cell aggregation, the plate-based CAT system effectively addresses these challenges [10,15,16]. By providing controlled conditions for cell aggregation, our approach ensures uniform particle formation and supports scalable chondrocyte production.

Chondrocyte-based regenerative therapies have been explored extensively as a means of treating defects in articular cartilage, particularly defects resulting from conditions, such as OA. Traditional methods, such as autologous chondrocyte implantation and osteochondral autograft transplantation are limited by the need for large quantities of chondrocytes, as well as the variability of therapeutic outcomes [2,3,4,5]. By contrast, the plate-based CAT system allows efficient production of chondrocyte particles that maintain high chondrogenic potential and can be readily applied to cartilage regeneration.

Histological and immunohistochemical analyses confirmed that the chondrocyte particles generated using the CAT system exhibited key markers of hyaline cartilage, including COL2 and ACAN, without expression of hypertrophic markers, such as COL1 and COL10. This indicates that the plate-based CAT-generated chondrocyte particles retain a stable cartilage phenotype, and are less likely to undergo undesirable hypertrophic differentiation, a common challenge for cartilage tissue engineers [17].

Furthermore, transplantation of plate-based CAT-generated chondrocyte particles into osteochondral defects in X-SCID rats demonstrated successful engraftment and cartilage regeneration. The defects were filled with cartilage-like tissue that stained positive for Safranin O and Toluidine Blue, indicating robust production of ECM. The transplanted particles were well integrated into the host tissue, as evidenced by the presence of human vimentin-positive cells within the repair site, suggesting that the plate-based CAT system may offer a viable strategy for clinical cartilage repair.

Despite these promising results, several challenges remain before the plate-based CAT system can be widely implemented in clinical settings. First, while the plate-based CAT system offers precise control over particle size and number, future studies should focus on optimizing the long-term stability and durability of in vivo cartilage tissue, including assessments of the mechanical strength of regenerated tissue and its integration with surrounding native cartilage. Additionally, although the CAT system simplifies the aggregation process, it may not fully replicate the complex three-dimensional microenvironment and biomechanical cues found in native cartilage. Modifications, such as scaffold-based or hydrogel supports, could better replicate the native cartilage environment.

An important consideration for clinical use is validation of large-scale production of chondrocyte particles under Good Manufacturing Practice (GMP) conditions to ensure safety and efficacy. To address these limitations, our future research will explore ways to enhance the CAT system’s capacity for large-scale production while meeting regulatory standards, and will also involve long-term in vivo studies to evaluate the functional durability of CAT-generated cartilage tissue in joint environments. By addressing these challenges, we aim to make the CAT system a clinically applicable solution for cartilage repair.

## 4. Materials and Methods

### 4.1. Cell Culture

The hiPSCs were maintained and cultured using StemFit (Ajinomoto, Tokyo, Japan, Cat# AK02N) according to protocols based on Yamada et al. [6]. Before reaching sub-confluence, the cells were dissociated using TrypLE Select (Thermo Fisher Scientific, Walthman, MA, USA)/0.25 mM EDTA and suspended in StemFit containing 10 μM Y27632 (FUJIFILM Wako, Osaka, Japan). A total of 1 × 10^4^ hiPSCs were then suspended in 2 mL of StemFit supplemented with 10 μM Y27632 and 8 μL of iMatrix511-silk (human laminin-511 E8 fragment, Nippi, Tokyo, Japan), and then seeded in a 6-cm dish. On the following day, the medium was replaced with fresh StemFit without Y27632, followed by medium changes every 2 days thereafter until the next passage. The 414C2 hiPSCs were obtained from the Center for iPS Cell Research and Application at Kyoto University, Japan.

For stepwise differentiation into LBM cells, hiPSCs (3 × 10^4^) were suspended in 1 mL of StemFit containing 10 μM Y27632 and 4 μL of iMatrix511-silk, and transferred to a 3.5-cm culture dish. On the following day, the medium was replaced with fresh StemFit without Y27632, and the hiPSCs were differentiated into mid-primitive streak, lateral plate mesoderm, and eventually into LBM cells, following established protocols [6].

For serial passage of ExpLBM cells, the LBM cells were dissociated with Accutase (Thermo Fisher Scientific, Walthman, MA, USA), and 2–4 × 10^5^ cells were resuspended in ExpLBM medium (CDM2 basal medium supplemented with 3 μM CHIR99021, 1 μM A-83-01, 20 ng/mL fibroblast growth factor 2, 20 ng/mL epidermal growth factor, 10 μM Y27632, and 1% penicillin-streptomycin (P/S; Gibco, Thermo Fisher Scientific, Waltham, MA, USA, Cat# 15140-122)). The cells were then cultured in 6 cm dishes coated with 4 μg/mL human plasma fibronectin (Merck, Darmstadt, Germany), followed by medium changes every 2 days. Passaging was performed before the cells reached sub-confluence, following the same protocol described above.

### 4.2. Immunocytochemistry

Cells cultured on dishes were fixed for 30 min at room temperature with 4% paraformaldehyde, followed by incubation for 1 h at room temperature in a blocking solution consisting of phosphate-buffered saline (PBS) containing 3% normal goat serum and 0.1% Triton X-100. The primary and secondary antibodies were diluted at 1:200 and 1:400–1:500, respectively, in blocking solution and then added to the cell cultures for 1 h at room temperature. After antibody incubation, nuclei were stained with 0.1 µg/mL of 4′,6-diamidino-2-phenylindole (DAPI; Thermo Fisher Scientific). Imaging was performed using a BZ-X710 fluorescence microscope (Keyence, Osaka, Japan). The primary antibodies used were anti-PRRX1 (Sigma-Aldrich, St. Louis, MO, USA, Cat# ZRB2165) and anti-SOX9 (Merck, Darmstadt, Germany, Cat# AB5535).

### 4.3. Flow Cytometry

Flow cytometric analysis was performed as described by Yamada et al. [6]. After enzymatic dissociation with Accutase, a cell suspension containing 1 × 10^5^ cells was prepared in 100 µL of PBS supplemented with 2% fetal bovine serum (FBS; Sigma-Aldrich, St. Louis, MO, USA, Cat# 173012). The cells were incubated on ice for 1 h with FITC-conjugated anti-CD90 and BB700-conjugated anti-CD140b antibodies, both diluted at 1:200. The CD90^high^CD140b^high^ population was analyzed using a CytoFLEX S flow cytometer (Beckman Coulter, Brea, CA, USA).

### 4.4. Fabrication of ExpLBM-Derived Chondrocyte Particle

A CAT plate (prevelex-CAT^®^; Nissan Chemical, Tokyo, Japan, Lot# IJ1D08KS1) was used for cell aggregation, (Figure 1a). This plate is coated with approximately 2300 polymer spots per well (24-well format), employing the CAT developed by Iwai et al. [11]. This system allows fabrication of a customizable number of cell aggregates. The CAT plate (prevelex-CAT^®^) was provided by Nissan Chemical Co. and features a surface coated with PDMAEMA-co-PMA-co-EGDM, which was dissolved in sterilized water at a concentration of 0.006% and applied onto a low-attachment culture vessel (PrimeSurface^®^; Sumitomo Bakelite, Tokyo, Japan) using an inkjet printer (LaboJet-Bio; Microjet, Tokyo, Japan), thereby forming numerous spots with a diameter of 250 µm. After drying at 70 °C for 24 h, the coating was sterilized by 25 kGy gamma-ray irradiation to ensure complete sterility. To induce chondrogenic differentiation under self-aggregation conditions, ExpLBM cells (2 × 10^5^) were suspended in 500 μL of ExpLBM medium and seeded onto a 24-well CAT plate (prevelex-CAT^®^).

After 2 days of culture in ExpLBM medium, the cell aggregates were treated for 6 days with Step 1 medium (CDM2 medium supplemented with 3 μM CHIR99021, 10 ng/mL FGF2, 50 μg/mL ascorbic acid, and 1× ITS) containing 10% FBS and 0.08% SphereMAX (Nissan Chemical, Tokyo, Japan). The floating cell aggregates were then transferred to Ultra-low dishes (PrimeSurface^®^; Sumitomo Bakelite, Tokyo, Japan) and washed with PBS before being treated for 5 days with Step 2 medium (CDM2 medium supplemented with 10 ng/mL FGF2, 50 μg/mL ascorbic acid, 30 ng/mL BMP4, 10 ng/mL TGF-β1, 10 ng/mL GDF5, and 1× ITS) containing 10% FBS and 0.08% SphereMAX. After this period, the cells were washed with PBS and replaced with Step 3 medium (CDM2 medium supplemented with 50 μg/mL ascorbic acid, 30 ng/mL BMP4, 10 ng/mL TGF-β1, 10 ng/mL GDF5, and 1× ITS) containing 10% FBS and 0.08% SphereMAX, and cultured for an additional 42 days. The differentiation medium was refreshed every 3 days during the culture period.

### 4.5. ExpLBM-Derived Chondrocyte Particles Transplantation

X-SCID rats (F344-Il2rgem1Iexas; The Institute of Medical Science, The University of Tokyo, Tokyo, Japan), aged 8–10 weeks, were used for the transplantation experiments. All experimental procedures and animal care protocols were approved by the Okayama University Animal Care and Use Committee (approval no. 2020814). Rats were housed two per cage, with free access to standard chow and water. Anesthesia was induced and maintained using isoflurane (4–5% for induction, 2–3% for maintenance) delivered via an inhalation anesthesia machine. A medial parapatellar incision was made on either the left or right knee, randomly assigned to each rat. Using a biopsy punch (Kai Industries, Gifu, Japan), the patella was moved laterally, and osteochondral defects (1-mm drill holes, 1-mm depth) were created in the patellofemoral groove. Marrow bleeding was observed during defect formation, confirming the osteochondral nature of the injury.

Rats transplanted with ExpLBM-derived chondrocyte particles were sacrificed by CO_2_ inhalation 4 weeks post-transplantation, depending on the experimental conditions. Tissue samples were fixed for 1 week in 10% formalin neutral buffer solution (FUJIFILM Wako, Osaka, Japan) and paraffin-embedded at the Central Research Laboratory of Okayama University Medical School. The in vivo cartilage tissue samples were decalcified with 10% EDTA-2Na prior to paraffin embedding to ensure proper tissue processing and sectioning.

### 4.6. Immunohistochemistry

Tissue samples were fixed in 10% formalin neutral buffer solution (FUJIFILM Wako, Osaka, Japan) or 4% paraformaldehyde and then embedded in paraffin. Sections (4 μm thick) were prepared from these paraffin-embedded samples. After deparaffinization, antigen retrieval was performed by heating the slides in 10 mM citrate buffer (pH 6.0). For specific antigens, such as COL1, COL2, COL10 and ACAN, an additional treatment with 1 μg/mL of Proteinase K was applied. Following antigen retrieval, the sections were incubated with a blocking solution containing 3% normal goat serum and 0.1% Triton X-100 in PBS for 1 h at room temperature.

Primary antibodies, diluted at 1:200, were applied overnight at 4 °C. After washing, the sections were incubated for 1 h at room temperature with fluorescent dye-conjugated secondary antibodies, diluted at 1:400. Both the primary and secondary antibodies were diluted in the blocking solution. After staining with DAPI, the sections were mounted using Fluoromount-G (SouthernBiotech, Birmingham, AL, USA), and images were captured using a BZ-X710 fluorescence microscope (Keyence, Osaka, Japan). The primary antibodies used were anti-hVIMENTIN (Progen Biotechnik, Heidelberg, Germany, Cat# 10515), COL1 (SouthernBiotech, Birmingham, AL, USA, Cat# 1441-01), COL2 (Thermo Fisher Scientific, Cat# MA1-37493), COL10 (Cosmo Bio, Tokyo, Japan, Cat# LSL-LB-0092), and ACAN (Proteintech, Rosemont, IL, USA, Cat# 13880-1-AP).

## 5. Conclusions

In summary, the plate-based CAT system is a novel and effective approach for generating chondrocyte particles from hiPSC-derived ExpLBM cells. This method holds significant potential for advancing cartilage regeneration therapies by providing a scalable and consistent source of chondrocytes. Moreover, future efforts will also aim to explore the potential of combining CAT-generated chondrocyte particles with other tissue engineering strategies, such as scaffold-based systems, to further enhance cartilage repair.

## Data Availability

All data generated during and/or analyzed during this study are included in the published article.
